# Enhancing Stirred Yogurt Quality With Hyaluronic Acid‐Rich Rooster Comb Extract: Effects on Texture and Shelf Life

**DOI:** 10.1002/fsn3.4666

**Published:** 2024-12-10

**Authors:** Nastaran Tabari Shahandasht, Marzieh Bolandi, Majid Rahmati, Moslem Jafarisani

**Affiliations:** ^1^ Department of Food Science and Technology, Damghan Branch Islamic Azad University Damghan Iran; ^2^ Department of Medical Biotechnology, School of Medicine Shahroud University of Medical Sciences Shahroud Iran; ^3^ Clinical Biochemistry Shahroud University of Medical Sciences Shahroud Iran

**Keywords:** dairy product, functional food, hyaluronic acid, hydrocolloid

## Abstract

Yogurt is a popular milk‐based product known for its nutritional benefits and effects on the large intestine. However, yogurt production faces challenges like texture, consistency, and syneresis. Hydrocolloids, such as gums and polysaccharides, can enhance yogurt's consistency and rheological properties. This research evaluates rooster comb extract (RCE) as a natural additive to improve stirred yogurt's properties during 21 days of storage at 4°C. Two treatments with 0.8 and 0.9 g of RCE were added to stirred yogurt. Results showed a decrease in pH (from 3.89 to 4.38) and microbial counts (> 10^7^ log CFU/g), along with an increase in titratable acidity (1.03%–1.48%) in RCE‐enriched yogurt (*p* < 0.05). The 0.8 g RCE treatment showed reduced syneresis, lightness, and setting time compared to the control (*p* < 0.05). Rheological analysis indicated thixotropic shear‐thinning behavior, accurately described by the Herschel–Bulkley model. All samples displayed solid viscoelastic properties, with the storage modulus exceeding the loss modulus in the linear viscoelastic region. While flavor and overall acceptability declined in enriched samples compared to controls (*p* < 0.05), no significant differences were found in other characteristics, including texture, color, and aroma (*p* > 0.05). In conclusion, RCE is a promising natural hydrocolloid for producing functional stirred yogurt, offering potential consumer benefits.

## Graphical abstract

A functional stirred yogurt was produced using two levels (0.8% and 0.9%) of rooster comb extract (RCE) successfully. Fortified yogurt during 21 days' storage at 4°C showed a significant decrease in syneresis, lightness, and setting time compared to control. Rheological properties exhibited thixotropic shear‐thinning behavior by the Herschel–Bulkley model. No significant difference was observed between treated and control samples in other organoleptic characteristics, texture, color, and aroma.

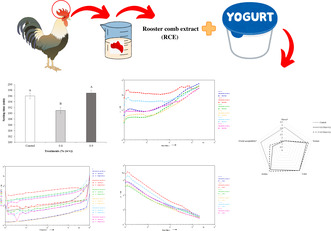

## 1 Introduction

Yogurt is a popular milk‐based product made by lactic acid bacteria (LAB). It is nutritious, and regular consumption may boost health. It affects metabolism in the large intestine. However, yogurt production can face issues like texture defects, consistency problems, and syneresis. Recently, natural modifiers like hydrocolloids have gained attention for dairy food fortification (Ozcan and Kurtuldu 2014; Xiong, Wen, et al. 2022). The fortification of milk products involves incorporating hydrocolloids into their structures (Xiong, Chen, et al. 2022). Hydrocolloids like gums, mucilage, and polysaccharides can enhance the consistency and strength of yogurt's gel‐like structure by binding to water molecules (Yang et al. 2020). Hydrocolloids enhance rheological attributes, which are crucial for designing pumps, selecting filters, and other processing variables. They also affect production costs and the organoleptic properties of the final product (Bakry, Chen, and Liang 2019). Many studies have been conducted to elucidate the mechanism of activity of hydrocolloid as stabilizers (Khubber et al. 2021; Pang et al. 2019), thickeners, gelling agents, and texture modifiers (Kashaninejad and Razavi 2019; Wong et al. 2020), individually or simultaneously. Reportedly, lower syneresis value and higher water‐holding capacity (WHC), viscosity, and modulus of elasticity in the gum‐enriched samples compared to the un‐enriched (control) one can diminish the negative effects resulted by less fat components. Furthermore, the results obtained indicate a significant correlation between the rheological parameters and sensory properties (Khubber et al. 2021; Wong et al. 2020). According to Pang et al. (2019) and Khubber et al. (2021), the microstructure of the enriched samples exhibited a homogeneous and uniform composition, in contrast to the control samples, which displayed a denser mass structure. This phenomenon is likely attributed to the numerous cross‐links formed in the enriched specimens, which may facilitate the filling of cavities. Additionally, Shi, Han, and Zhao (2017) and Tobil et al. (2020) suggested that the incorporation of cross‐linked gelatin and pectin in yogurt can moderately delay post‐acidification, inhibit aerobic microbial growth, and diminish protein aggregate sedimentation during storage.

Hyaluronic acid (HA) or hyaluronan is a nonsulfated, linear, anionic, heteropolysaccharide (Hamad et al. 2017) consisting of repetitive units of β1.4‐d‐glucuronic acid and β1.3‐*N*‐acetyl‐d‐glucosamine (Pourzardosht and Rasaee 2017; Zakeri, Rasaee, and Pourzardosht 2017). This compound has a molecular weight of 10^4^–10^7^ Da. It belongs to the glycosaminoglycan family, which is present in almost all tissues and biological fluids. HA is produced during the fermentation of LAB, i.e., *Streptococcus thermophilus* (Martinez‐Puig et al. 2013), which is believed as a safe, and green technology (Hamad et al. 2017). Commercially, this extracellular polysaccharide is extracted from the mucoid layer of rooster comb (RC), considered the most accessible source of high‐molecular‐weight HA. Additionally, rooster comb extract (RCE) has high hydrophilicity and viscosity due to HA (Hamad et al. 2017). On the other hand, using RC in food industries can reduce abattoir waste and lower costs. Rooster comb extract (RCE) is rich in glycosaminoglycans, minerals, vitamin D, edible fibers, free amino acids, and antioxidants, enhancing its nutritional value (Hafsa et al. 2017; Mohammed and Niamah 2022; Ramos‐Peralonso 2013). The enrichment of the stirred yogurt with RCE might reduce fat intake, control weight in osteoarthritis patients (Martinez‐Puig et al. 2013), and promote the physicochemical, textural, and sensory properties of the RCE‐containing dairy food product. Chon et al. (2020) reported that supplementing kefir with hyaluronic acid (HA) enhanced both its physicochemical properties and organoleptic features. Kefir granules can break down milk proteins into small bioactive peptides with health benefits. These peptides serve as a nitrogen source for lactic acid bacteria (LAB), enabling the production of more HA chains under suitable fermentation conditions. However, there is limited information in the literature about various HA‐added fermented milk products.

The objective of this research was to evaluate the use of rooster comb extract (RCE) as a natural additive to improve the functional properties of stirred yogurt during 21 days of storage.

## 2 Materials and Methods

The rooster comb was purchased from the poultry slaughterhouse (Amol, Iran). Skim milk (Cat. No. 1.15363; Merck, Darmstadt, Germany) (pH 6.78, acidity: 13.05%, fat: 0.05%, protein: 3.2%, total dry matter: 8.42%) and skim milk powder (70166; Sigma‐Aldrich, Germany) (fat: 0.6%, protein: 38.7%, total dry matter: 98.35%) were used with standard milk composition. Direct starter culture (a mixture of 1:1 *Streptococcus thermophilus* and *Lactobacillus bulgaricus* ) was purchased from the Christian Hansen's company, Denmark. All the chemicals used were of analytical reagent grade.

### 2.1 Extraction and Purification Procedure of HA

The extraction and purification of hyaluronic acid (HA) followed the methods of Khabarov, Boykov, and Selyanin (2015) and Mozzi et al. (2006) with minor modifications. A total of 50 g of precleaned, blood‐drawn RC tissue was mixed with 500 mL of distilled water (DW) and incubated at 60°C for 30 min in a conventional incubator (INC 108; Memmert GmbH, Germany). A physiological saline solution (250 mL) was then added, and the mixture was kept at 80°C for 1 h. The extract was filtered through a cotton cloth and centrifuged (Model SW14R‐Friolabo, France) at 2800 ×g for 10 min. The resulting extract was mixed with 100 mL of ethanol (Merck) and stored at 4°C for 1 day to separate impurities; ethanol was added to remove impurities, resulting in no residue in the obtained HA. The isolated precipitate (pH 6.75; dry matter content, 1.37%; extraction yield, 1.26%; HA content, 4.19 mg/g) was stored in a dark sterile container in the refrigerator until further testing.

### 2.2 Preparation of the Incorporated Stirred Yogurt Samples

First, skim milk powder was used to standardize the dry matter of milk. Fresh milk, pasteurized at 85°C for 30 min and cooled to 45°C, was inoculated with a 2% (w/v) starter culture while being stirred uniformly. The inoculated milk was then incubated at 43°C (Memmert Incubator, INB 400; Memmert GmbH) until a pH of 4.61 was reached, after which it was stored overnight at 4°C (Tobil et al. 2020). To obtain enriched stirred yogurt, two concentrations of RCE at 0.8 and 0.9 g were added to 100 mL of preheated and inoculated milk and thoroughly mixed. These concentrations were chosen based on the maximum allowable daily consumption of RCE (Ramos‐Peralonso 2013) and preliminary treatment (physicochemical and rheological experiments) results. One sample was designated as the control group (without RCE). All samples were stored at 4°C in a dark container for physicochemical analyses on days 1, 7, 14, and 21. Sensory evaluations were conducted on the 21st day of cold storage. The yogurt production and enrichment procedure were carried out twice (two replicates) for all analyses (Tobil et al. 2020).

### 2.3 Measurement of pH and Titratable Acidity

The pH of the treated samples was measured using a calibrated pH meter (Orion model 0290; Thermo Scientific) and probe (VWR model 89231‐572; VWR Analytical) (AOAC 2005). Titratable acidity was evaluated using the protocol of AOAC (2005). The sample (2.5 g) and 25 mL of DW were poured in a beaker. Then, titration was employed by 0.1 N NaOH. The titratable acidity was defined as a percentage of lactic acid.

### 2.4 Syneresis Index

Whey loss was estimated using the method of Ye et al. (2013). Twenty‐five grams of yogurt was centrifuged (Eppendorf 5810R, Chennai, India) for 20 min at 1000 × g. The syneresis value was calculated by dividing the weight of the sample after centrifugation by the total sample weight. The results were reported as a percentage.

### 2.5 Microbial Analysis

The method of Demirci et al. (2020) was used for determining the viable number of LAB in the yogurt samples. A 1 g of yogurt was diluted with a sterile Ringer solution (1:10) serially. Two dilutions, 10^−6^ and 10^−7^, were cultured on petri dishes containing sterile MRS agar medium (Merck) in triplicate. The plates were incubated at 37°C ± 1°C for 72 h. The bacterial colonies developed after incubation (Memmert Incubator, INB 400; Memmert GmbH) were expressed as colony‐forming units per gram (log CFU/g) (Guo et al. 2024).

### 2.6 Color Determination

The color parameters of the yogurt samples (2 g) were analyzed using a colorimeter (Precision Colorimeter, Model NR 145, China) with the CIELAB system. The parameters L*, a*, and b* were measured, representing brightness from black (0) to white (100), redness and greenness from +120 to −120, and yellowness and blueness in similar ranges. Before the experiment, the device was calibrated, and the ambient temperature was adjusted to 23°C–24°C for the samples (Yekta and Ansari 2019).

### 2.7 Setting Time

The procedure by Yousef and Rusli (1995) was used to record time in minutes. Samples were incubated at 42°C ± 1°C, and coagulum formation was monitored every 30 min by measuring pH values (Orion model 0290; Thermo Scientific). The setting period was calculated from the start of incubation until the pH of the coagulated sample reached 4.60–4.70.

### 2.8 Rheological Properties

The method of Pang et al. (2019) was used to measure the rheological properties of the stirred yogurt specimens with a rheometer (MCR 502; Anton Paar GmbH, Graz, Austria) equipped with a parallel steel plate geometry (PP60; 60 mm diameter and 1 mm gap setting) at 20°C. The tests included flow behavior and dynamic viscoelastic properties. For each test, 3 mL of the yogurt gel was loaded into the rheometer, and measurements were taken on the initial and final days of storage (1 and 21).

#### 2.8.1 Flow Behavior Test

Flow behavior test was performed to recognize the changes in shear stress (*τ*) and apparent viscosity (*η*), in the shear rates of 10^−1^–10^−3^ s^−1^. Besides, to determine the appropriate model to describe the flow behavior, the power law, Herschel–Bulkley, and Carreau models were compared, and their parameters were specified. The TA Instruments Trios v4.2.1 data analysis package was applied to calculate the parameters.

(1) $$\tau =m\;\gamma^{\cdot n}$$

(2) $$\tau =m\gamma^{\cdot n}+\tau_{0}$$

(3) $$\left(\eta \left(\gamma^{\cdot }\right)-\eta_{\infty }\right)/\left(\eta_{0}-\eta_{\infty }\right)={\left[1+{\left({\lambda \gamma }^{\cdot }\right)}^{a}\right]}^{n-1/a}$$

Equations ((1), (2), (3)) are related to the power law, the Herschel–Bulkley, and the Carreau models, respectively. In the abovementioned equations, *τ*_0_, *γ*˙, *m*, and *n* refer to yield stress, shear rate, consistency coefficient, and flow behavior index, respectively. Also, in the third equation, the fixed values of *η*_∞_, *η*_0_, *n*, λ, and *a* are the viscosities at infinite and zero shear rates, the power index, the time constant, and a dimensionless constant, respectively (Pang et al. 2019).

#### 2.8.2 Dynamic Viscoelastic Properties

The frequency sweep test was carried out from the frequency of 0.01–100 Hz at a constant strain of 0.1% within the linear viscoelastic region of the specimens. Parameters obtained from the oscillatory tests consist of the dynamic modulus, i.e., storage modulus (*G*′), and the loss modulus (*G*″). In addition, the complex viscosity (η*) and the loss tangent (tan *δ*) were monitored on the initial and final days of cold storage. The *G*′ and *G*″ moduli were reported as a function of frequency. η* was computed from the ratio of the complex modulus to the frequency, while tan *δ* was calculated from the ratio of *G*″ to *G*′ (Kashaninejad and Razavi 2019).

### 2.9 Sensory Evaluation

Sensory properties of all the specimens were determined using a 5‐point hedonic scale based on the instructions suggested by Tobil et al. (2020). Twelve trained panel members evaluated organoleptic attributes, such as texture, color, flavor, aroma, and overall acceptance, randomly. They then scored them from 1 to 5, with 1 assigned to “dislike very much” and 5 to “like very much,” respectively. Distilled water was used to remove the residual flavor of the samples.

### 2.10 Statistical Analysis

First, the normal distribution of the variables was verified before analysis. Then, the effects of enrichment and storage time as well as their interaction were investigated on the responses through the repeated measures procedure. All parameters were measured in triplicate except for the sensory properties, which had 12 replications, and all the experiments were performed twice. The data were analyzed using IBM SPSS (version 25.0; SPSS Inc., Chicago, IL, USA) using analysis of variance (ANOVA) and Duncan's multiple range test at a 95% confidence level.

## 3 Results and Discussion

### 3.1 Effect of RCE on Physicochemical and Microbial Attributes

Table 1 shows the changes in pH and titratable acidity values of all samples over 21 days of storage. The sample with 0.8 g RCE showed a significant decrease in pH compared to other treatments. A significant reduction was observed on the 7th and 14th days after enrichment compared to the 21st day. On the 21st day, the mean pH value of the 0.8 g RCE treatment was the highest, with no significant differences between treatments containing 0.9 g RCE and the un‐enriched group. This pH increase is apparently associated with the higher concentration of the nitrogen components generated during proteolysis over storage (Bulut, Alwazeer, and Tunçtürk 2023). The pH of the control treatment reduced after 7 days. During storage, titratable acidity followed a rising trend, with the sample containing 0.8 g RCE showing a significantly higher acidity (*p* < 0.05) after 14 days compared to the control and 0.9 g RCE samples. No significant differences were observed between control and 0.9 g RCE samples (*p* > 0.05) on the 14th and 21st days. However, the 0.8 g RCE sample showed significant changes in acidity from days 14 to 21 (*p* < 0.05). The increase in acidity is primarily due to lactic acid production by LAB, which is enhanced by the polysaccharides and prebiotic features of RCE, leading to a decrease in pH. Meanwhile, due to having a low decomposition factor in aqueous media, weak organic acids, i.e., glucuronic acid in RCE, break down slightly, producing H^+^, which can lower the pH and increase the acidity of enriched samples. In line with these findings, Sendra et al. (2010) claimed that the presence of citric fiber in the fermented milk products may increment the rate of growth and viability of starters in yogurt, thereby leading to the conversion of lactose into lactic acid more quickly. Also, Ladjevardi, Gharibzahedi, and Mousavi (2015), Arioui, Ait Saada, and Cheriguene (2017), and Chon et al. (2020) confirmed previous outcomes about the role of arabinoxylan in husk gum, pectin from citrus peel, jujube mucilage, and liquid HA, respectively. These substances included complex polysaccharides participating in fermentation as well as speeding up the reactions which led to a higher acidity percentage in the fortified yogurt. In contrast, Hassan et al. (2015) stated that the changes in the pH and acidity conditions were nonsignificant when the yogurt specimens were fortified with a concentration of 1% guar gum.

**TABLE 1:** Effect of enrichment with HA extracted from rooster comb on the physicochemical and microbial properties of the stirred yogurt during storage at 4°C.

Attribute	Treatment (% (w/v))	Storage time (day)			
		1	7	14	21
pH	Control	4.41 ± 0.21^Aa^	4.38 ± 0.11^Aa^	4.03 ± 0.12^Bc^	4.27 ± 0.05^Bb^
	0.8	4.21 ± 0.13^Bb^	4.00 ± 0.13^Cc^	3.89 ± 0.20^Cd^	4.38 ± 0.02^Aa^
	0.9	4.37 ± 0.12^Aa^	4.20 ± 0.00^Bbc^	4.13 ± 0.13^Ac^	4.24 ± 0.03^Bb^
Titratable acidity (%)	Control	1.03 ± 0.01^Bc^	1.20 ± 0.02^Cc^	1.47 ± 0.01^Ba^	1.45 ± 0.02^Aab^
	0.8	1.17 ± 0.02^Ac^	1.48 ± 0.01^Aa^	1.51 ± 0.03^Aa^	1.38 ± 0.04^Bb^
	0.9	1.14 ± 0.03^Ac^	1.40 ± 0.03^ABa^	1.42 ± 0.05^BCa^	1.48 ± 0.02^Aa^
Syneresis index (%)	Control	54.67 ± 3.48^Ab^	60.34 ± 4.51^Aa^	60.47 ± 5.21^Aa^	60.53 ± 4.58^Aa^
	0.8	52.85 ± 3.25^Ac^	53.30 ± 3.02^Bb^	53.8 ± 4.05^Bb^	55.26 ± 2.52^Ba^
	0.9	42.8 ± 2.08^Bc^	47.72 ± 3.18^Cb^	48.81 ± 3.20^Cb^	54.12 ± 2.36^Ba^
Microbial counts (log CFU/g)	Control	8.80 ± 0.64^Aa^	7.57 ± 0.53^Bb^	7.35 ± 0.57^Bc^	7.20 ± 0.63^Cc^
	0.8	8.88 ± 0.46^Aa^	7.74 ± 0.62^Ab^	7.50 ± 0.40^Ac^	7.55 ± 0.45^Ac^
	0.9	8.83 ± 0.50^Aa^	7.66 ± 0.18^ABb^	7.42 ± 0.27^Ac^	7.39 ± 0.28^Bc^

*Note:* Values are mean ± standard deviation in triplicate. A–D: Different letters within the supplemented treatments at the same time (columns); a–d: Different letters within the storage time (rows) indicate statistically significant differences at the probability level of 95%.

Notably, syneresis in yogurt signifies the separation of whey from the gel‐like structure of yogurt. It is also known as a time‐dependent parameter associated with WHC. Several factors such as pH, acidity, temperature, duration of storage, fat, polysaccharide, and protein contents affect the WHC. Obviously, syneresis is inversely influenced by the WHC of the protein network. The more the WHC, the less syneresis is. Besides, fortifying yogurt with hydrocolloids like gums, mucilage, and dietary fibers could modify its structural defects due to their capacity to absorb and retain water (Hassan et al. 2024). The occurrence of discrepancies in whey drainage of stirred yogurt varied over the storage period, as demonstrated in Table 1. The rate of whey separation significantly (*p* < 0.05) increased during cold storage, peaking on the 21st day. This rise is attributed to the proteolytic activity of LAB, which varied in samples with or without RCE. The polysaccharides in RCE enhanced LAB activity, leading to increased proteolysis and acidity, supporting the pH and acidity results from the study. Yekta and Ansari (2019) demonstrated a strong correlation between the activity of starter bacteria and acidity (*R* ^2^ > 0.97). As the activity of starter bacteria increased, both acidity and proteolysis rates elevated. Consequently, proteins retained less water, as evidenced by the rising trend of syneresis over time in all samples. On the initial day of storage, no significant changes were observed between the plain sample (0% RCE) and the 0.8 g RCE treatment (*p* > 0.05, Table 1), but differences emerged with longer storage. The addition of RCE resulted in significant differences in whey separation across all treatments from the 7th day of storage onward (*p* < 0.05). By the last day of storage, the control group exhibited the highest syneresis index, while the lowest values were observed in the 0.9 and 0.8 g RCE samples. No significant changes in syneresis mean values were found between the enriched samples (*p* > 0.05, day 21). RCE reduces whey separation by increasing the viscosity (*η*) of the continuous phase. The hyaluronic acid (HA) in RCE can bond with free water, leading to higher *η* and lower syneresis compared to the control group. These changes may be linked to the structural rearrangements of proteins and modifications of protein–protein linkages that form new connections with HA. Additionally, the increased total solid content of milk helps prevent the separation of free serum in fortified samples with RCE. The synergistic effect of RCE and starter culture bacteria, which produce exopolysaccharides, can enhance water‐holding capacity (WHC), delay whey separation, and alter yogurt texture. Khubber et al. (2021) found that adding water‐soluble polysaccharides to low‐fat yogurt could move water molecules within the protein gel network, leading to reduced whey depletion. This gel network reinforcement occurred due to the negatively charged carboxyl groups in hyaluronic acid (HA), which bonded with the positively charged casein whey protein. Some researchers have reported remarkable reduction in the release of whey after supplementing with hydrocolloids such as cress seed mucilage or guar gum (Hassan et al. 2015), modified starch (Pang et al. 2019), jujube mucilage (Yekta and Ansari 2019), low‐methoxyl pectin (Khubber et al. 2021), and apple pomace (Wang, Kristo, and LaPointe 2020). Moreover, some reported a remarkable increase in syneresis by adding guar gum (Bahrami et al. 2013), disaccharides (Nazan 2022), and applying ultrasound (Körzendörfer and Hinrichs 2019) in yogurt manufacturing.

As shown in Table 1, microbial counts in stirred yogurt were significantly affected by fortification, time, and their interaction. The viability of starter bacteria significantly (*p* < 0.05) decreased over time, starting from the initial day of storage and becoming pronounced by the 7th day. However, the number of viable cells remained relatively unchanged from the 14th to the 21st day of cold storage, indicating no significant (*p* > 0.05) changes. The addition of RCE, which contains hyaluronic acid (HA) polymer, can reduce the required water activity for the growth of starter bacteria, leading to decreased starter viability. This reduction may lower the production of flavor compounds such as diacetyl and acetaldehyde (Yekta and Ansari 2019). Therefore, these findings align with the results of the sensory analysis (ultimate section), which indicated lower palatability in the RCE‐enriched treatments compared to the control sample. Additionally, higher bacterial viability may have led to increased organic acids and, consequently, higher acidity in RCE‐added yogurts, adversely affecting microbial viability. Higher bacterial counts were observed in fortified yogurt samples compared to the unfortified control (*p* < 0.05, Table 1), highlighting the role of saccharide fractions forming HA chains as a carbon source for the growth of LAB. The results of this experiment (> 10^7^ log CFU/g viable cells) are consistent with previously recorded data (Khubber et al. 2021). As reported by Arioui, Ait Saada, and Cheriguene (2017), an exponential increment in starter population over time exhibited a direct proportional relationship between storage time and microbial load, and the rate of pectin as a carbon resource was added in yogurt. Similar findings have been reported by Demirci et al. (2020), Huang et al. (2020), and Khubber et al. (2021) on the enhancement of the growth and survival rate of bacteria in yogurts incorporated with tomato powders, polydextrose, and low‐methoxyl pectin, respectively.

### 3.2 Color Properties

From the consumer's perspective, color is a key quality feature in dairy products, reflecting freshness and flavor. The color parameters of the fortified samples were influenced by both time and stabilizer concentration (*p* < 0.05, Table 2). As summarized in Table 2, the L* value of all samples significantly increased, while a reverse trend occurred for the a* and b* values over time (*p* < 0.05, at 1–21 days). Extended cold storage of yogurt samples (with or without RCE) led to increased lightness and reduced redness and yellowness. These changes may be related to the decomposition of carbon sources, such as HA in treated samples, and its utilization by LAB over 21 days of storage. The observed reduction in pH during the fermentation process supports these findings. Adding RCE resulted in a noticeable (*p* < 0.05) reduction in brightness compared to the group without RCE. Additionally, the incorporation of RCE into the stirred yogurt formula significantly (*p* < 0.05) increased red or green (a*) and yellow or blue (b*) pigments compared to the control group. The negative values of a* and positive values of b* indicate the greenish and yellowish hues of the respective treatments. The interaction between polysaccharides in RCE and milk proteins, such as whey proteins and casein micelles, rearranges these macromolecules, resulting in more compact structures. These alterations can affect light reflection, giving the specimens a distinctive appearance (Ghosh et al. 2024). On the other hand, the addition of RCE, which refers to the binding capacity of HA, reduced the free water content, resulting in darker samples. Furthermore, as previously discussed, incorporating RCE into the yogurt samples decreased syneresis, leading to less separation of the riboflavin vitamin. Although the samples appear white to the human eye, the pigment of RCE‐added samples, as determined by instrumental systems, tends to fall within the green color range. Notably, no significant differences (*p* > 0.05, 1–14 days) were observed in color parameters between the 0.8 and 0.9 g treatments, indicating their comparable effects in this regard. Ozcan and Kurtuldu (2014) also documented the elastic matrix of casein–protein–β glucan, which corresponds to the negative and positive values for a* and b* parameters, respectively, in probiotic yogurt containing dietary fiber.

**TABLE 2:** Effect of enrichment with HA extracted from rooster comb on color parameters of the stirred‐type yogurt during storage at 4°C.

Attribute	Treatment (% (w/v))	Storage time (day)			
		1	7	14	21
L*	Control	80.95 ± 3.05^Ac^	81.38 ± 5.09^Ab^	82.65 ± 3.00^Aa^	82.68 ± 3.56^Aa^
	0.8	80.67 ± 3.25^Bc^	80.81 ± 4.15^Bc^	81.94 ± 3.13^Bb^	82.46 ± 3.11^Ba^
	0.9	80.27 ± 3.19^Bc^	80.63 ± 4.01^Bc^	81.91 ± 3.20^Bb^	82.37 ± 4.20^Ba^
a*	Control	−1.40 ± 0.04^Ca^	−1.55 ± 0.01^Bb^	−1.78 ± 0.06^Cc^	−1.88 ± 0.07^Bd^
	0.8	−1.06 ± 0.05^Aa^	−1.12 ± 0.03^Ab^	−1.39 ± 0.02^Ac^	−1.59 ± 0.01^Ad^
	0.9	−1.01 ± 0.02^Ba^	−1.28 ± 0.00^Ab^	−1.37 ± 0.06^Ac^	−1.57 ± 0.07^Ad^
b*	Control	11.52 ± 1.14^Bd^	10.45 ± 1.17^Bc^	9.73 ± 0.26^Bb^	8.67 ± 0.23^Ca^
	0.8	12.20 ± 2.41^Ad^	11.35 ± 0.13^Ac^	10.41 ± 0.19^Ab^	9.89 ± 0.44^Aa^
	0.9	12.88 ± 2.25^Ad^	11.34 ± 0.09^Ac^	10.51 ± 0.67^Ab^	9.45 ± 0.73^Ba^

*Note:* Values are mean ± standard deviation in triplicate. A–D: Different letters within the supplemented treatments at the same time (columns); a–d: Different letters within the storage time (rows) indicate statistically significant differences at the probability level of 95%.

### 3.3 Effect of RCE on Setting Time

**FIGURE 1:**
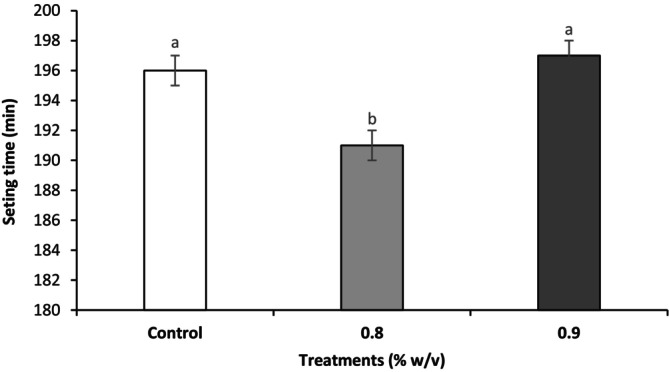
Effect of enrichment with HA extracted from rooster comb on the setting time of the stirred yogurt. Different lowercase letters indicate statistically significant differences at the probability level of 95%.

Figure 1 shows the coagulation time of functional yogurt supplemented with two concentrations of RCE. The effect of RCE on the setting time was statistically significant (*p* < 0.05); the coagulation time was significantly decreased in the 0.8 g RCE treatment (191 min) compared to other samples. However, there were no significant differences (*p* > 0.05, Figure 1) between the mean coagulation times for the 0.9 g RCE (197 min) and control group (196 min). This observation may be attributed to the intrinsic pH of RCE at the time of inoculation into milk and the enhanced metabolism of starter cultures under the favorable nutritional and environmental conditions created by RCE. This results in a rapid drop in pH values and an increase in acidity during fermentation, requiring less time for gel setting. Additionally, the synergistic effect of yogurt starters with RCE reduced the fermentation duration in this study. RCE is a rich source of macro‐ and micronutrients, particularly polysaccharides (Hafsa et al. 2017) that provide optimal nutritional conditions for the activity of LAB, explaining the production of more organic acids, i.e., lactic and acetic acids during fermentation. These changes accelerate the fermentation rate, and faster coagulation occurs. Indeed, reducing the setting time consumes less energy, so it is directly related to lowering the production costs (Bakry, Chen, and Liang 2019). Similar findings have been reported in shortening the coagulation time after supplementing milk‐base materials with passion fruit peel powder (Do Espírito Santo et al. 2012) and whey protein concentration (Glušac et al. 2015).

### 3.4 Effect of RCE on Rheological Properties During Storage

**FIGURE 2:**
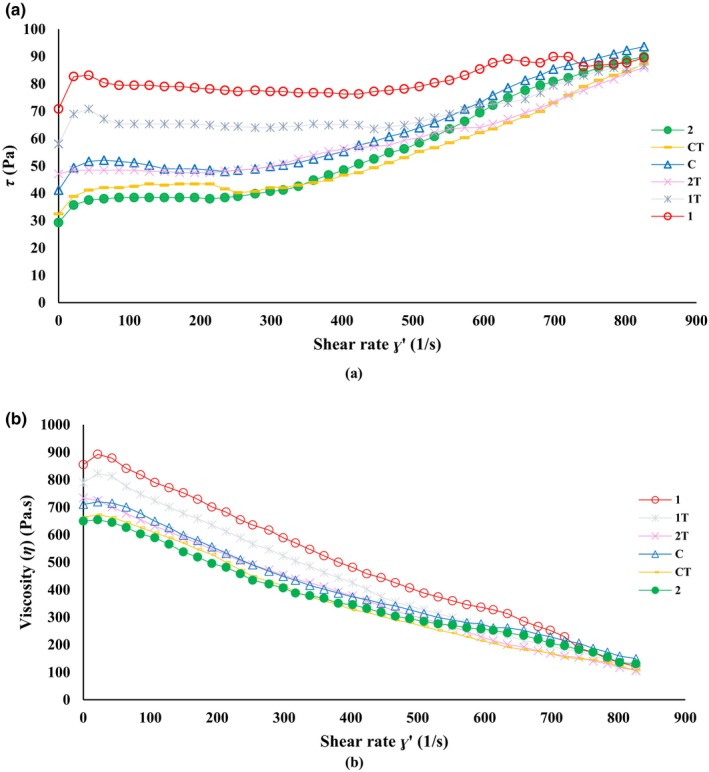
Effect of enrichment with HA extracted from rooster comb on *τ* = shear stress (a) and *η* = viscosity (b) of the samples during storage at 4°C. Treatments—C: control, 1: the stirred yogurt containing 0.8 g RCE, 2: the stirred yogurt containing 0.9 g RCE on the 1st day of storage; CT: control, 1T: the stirred yogurt containing 0.8 g RCE, 2T: the stirred yogurt containing 0.9 g RCE on the 21st day of storage.

The effect of yogurt enrichment on the flow curve and its parameters (*τ* and *η*) in relation to *γ*˙ is shown in Figure 2a,b. Figure 2a indicates a nonlinear relationship between *τ* and *γ*˙, classifying the system as non‐Newtonian fluids. As *γ*˙ increases, *η* of the samples decreases (Figure 2b), indicating shear‐thinning behavior. In gel‐type structures like stirred yogurt, molecules are irregularly arranged and partially aligned at low *γ*˙, leading to higher *η*. As *γ*˙ increases, molecular alignment improves in the shearing direction, resulting in increased internal friction and decreased *η*. This reduction in *η* with increasing *γ*˙ can be attributed to smaller colloidal masses and potential breakdown of polymers in the treated samples. Previous studies have shown that low *γ*˙ affects food consistency, while *η* emerges at larger *γ*˙ (Abedinia et al. 2017; Yekta and Ansari 2019). Additionally, samples containing 0.8 g of RCE exhibited higher *η* than others on both days of storage (1 and 21), likely due to changes in protein–protein and protein–polysaccharide connections, as well as HA molecules' water absorption. Morell et al. (2015) noted that the higher water‐binding capacity of hydrocolloids reduces flow ability, thereby increasing *η*. Conversely, the lower *η* in the 0.9 g RCE treatment is likely due to reduced intermolecular interactions in the continuous phase. In fermented products like yogurt, this rheological behavior results from weak electrostatic and hydrophobic interactions (Kashaninejad and Razavi 2019; Pang et al. 2019). The collapse of these structures may lead to decreased *η*, as observed in the 0.9 g RCE treatment.

Table 3 presents the parameters from the power law, Herschel–Bulkley, and Carreau models for HA‐fortified yogurt over 21 days of storage. According to the coefficient of determination (*R* ^2^ > 0.90), both the Herschel–Bulkley and Carreau models fit the experimental data well for all samples. However, given the flow index for pseudoplastic fluids (0 < *n* < 1), the Herschel–Bulkley model more accurately describes the rheological behavior of the stirred yogurt samples (Azari‐Anpar et al. 2017). The thixotropic behavior of the yogurt samples was observed over time, with a reduction in viscosity (*η*) at higher shear rates (*γ*˙) in the RCE‐treated specimens. In thixotropic fluids, structural rebuilding occurs after the stress is removed, indicating a weak gel structure in the yogurt. This suggests that in the treated samples, reconstructing the protein network into a cohesive structure after shearing is more challenging compared to untreated yogurts (Cui et al. 2014). In this research, the HA‐treated yogurt samples displayed distinct rheological behaviors, with lower flow index (*n*) and consistency index (*m*) values compared to the control, confirming their shear‐thinning and thixotropic nature (*p* < 0.05). The presence of HA in RCE significantly impacted key parameters, such as yield stress (*τ*_0_), *n*, and *m* (*p* < 0.05, Table 3). The higher *τ*_0_ values in the fortified samples indicated that more stress was needed to initiate flow compared to the control (*p* < 0.05). These rheological parameters are closely linked to sensory characteristics and consumer preferences, which will be discussed later (Harte, Clark, and Barbosa‐Cánovas 2007). These outcomes were consistent with the results of Paseephol, Small, and Sherkat (2008), Azari‐Anpar et al. (2017), and Pang et al. (2019).

**TABLE 3:** Effect of enrichment with HA extracted from rooster comb on rheological parameters of the power law, Herschel–Bulkley, and Carreau models of the stirred‐type yogurt during storage at 4°C.

Storage time (day)	Treatments (% (w/v))	Power law model			Herschel–Bulkley model				Carreau model		
		*τ*_0_ (pa)	*m* (pa·s^*n*^)	*R* ^2^	*τ*_0_ (pa)	*m* (pa·s^*n*^)	*n*	*R* ^2^	*τ*_0_ (pa)	*n*	*R* ^2^
1	Control	11.49^A^	0.304^B^	0.88	7.75^B^	2.14^C^	0.68^A^	0.96	0.38^B^	−0.25^B^	0.98
	0.8	7.33^C^	0.371^A^	0.93	33.24^A^	2.30^A^	0.49^C^	0.94	0.17^C^	−0.09^A^	0.90
	0.9	7.75^B^	0.287^C^	0.82	4.08^C^	2.21^B^	0.65^B^	0.97	0.67^A^	−0.26^C^	0.98
21	Control	7.18^C^	0.29^A^	0.84	5.64^C^	1.11^B^	0.72^B^	0.99	0.29^B^	−0.26^C^	0.99
	0.8	19.49^A^	0.09^C^	0.61	18.69^A^	0.53^C^	0.82^A^	0.92	0.07^C^	−0.19^B^	0.95
	0.9	9.85^B^	0.24^B^	0.89	8.13^B^	1.64^A^	0.62^C^	0.99	0.44^A^	−0.18^A^	0.97

*Note:* Means are compared only within a column; mean values with different capital letters have statistically significant difference at the probability level of 95%.

**FIGURE 3:**
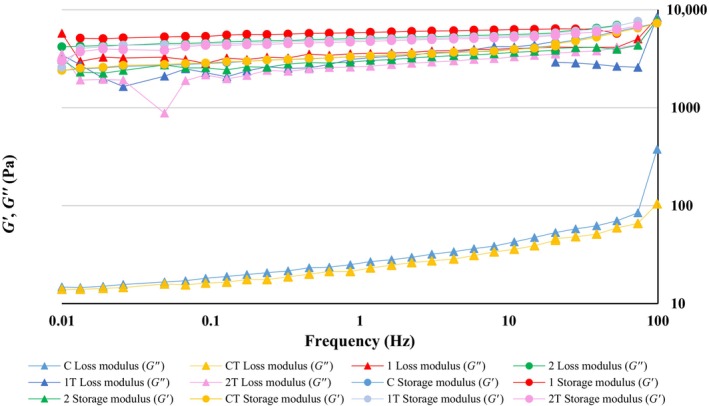
Effect of enrichment with HA extracted from rooster comb on dynamic modulus (*G*′, *G*″) of the samples during storage at 4°C. Treatments—C: control (blue), 1: the stirred yogurt containing 0.8 g RCE (red), 2: the stirred‐type yogurt containing 0.9 g RCE (green) on the 1st day of storage; CT: control (yellow), 1T: the stirred yogurt containing 0.8 g RCE (light blue), 2T: the stirred yogurt containing 0.9 g RCE (pink) on the 21st day of storage.

The rheological behavior of the gel‐like yogurt structure, explained through the *G*′ (elastic modulus) and *G*″ (viscous modulus) parameters, was affected by the incorporation of RCE. As shown in Figure 3, both *G*′ and *G*″ values increased with frequency, showing a gentle upward slope. Yogurt samples enriched with 0.8 and 0.9 g of RCE exhibited higher dynamic modulus than the control group on days 1 and 21 of storage. *G*′ values, being higher than *G*″ across all samples, emphasized the viscoelastic and solid‐like characteristics of the yogurt, likely due to the strengthening effect of HA chains within the protein network. This solid‐like behavior supports the previously discussed flow curve findings. Yekta and Ansari (2019) demonstrated that adding jujube mucilage to stirred yogurt increased its dynamic modulus due to interactions between casein micelles and hydrocolloids. In this study, the frequency sweep test results at 1 Hz (Table 4) show that the sample containing 0.8 g of RCE exhibited significantly higher *G*′, *G*″, and η* values compared to both the control and the yogurt with 0.9 g RCE on the first day (*p* < 0.05). Over time, these viscoelastic parameters in the sample with a higher RCE concentration increased significantly (*p* < 0.05) compared to the control. The *G*′ values being greater than *G*″ throughout the storage period (*p* < 0.05) indicated a stronger, solid‐like gel structure in the RCE‐enriched yogurts, linked to the strength of covalent and noncovalent bonds in the casein network and their degradation at higher shear rates (Sutariya and Salunke 2022). The η* values, reflecting sample stiffness, indicated that the addition of RCE significantly enhanced (*p* < 0.05, Table 4) the strength and resistance to deformation of the yogurt structure compared to those without RCE. This suggests that the RCE‐treated samples formed a more integrated and stable gel structure. The presence of HA molecules in RCE contributed to a more rigid texture in the yogurt. Similar increases in η* values have been reported in yogurts fortified with orange fiber, which also improved the yogurt's structural integrity (Sendra et al. 2010). Furthermore, tan *δ* represents the ratio of dissipated (viscous) energy to stored (elastic) energy, indicating the balance between the viscous and elastic properties of food. A tan *δ* value > 1 suggests that the sample exhibits more viscous, liquid‐like behavior, whereas a value < 1 indicates predominant elasticity and a solid‐like behavior (Kashaninejad and Razavi 2019). In this trial, tan *δ* values of all samples were < 1 (*p* < 0.05), verifying greater contribution of the elastic ingredients to the viscoelasticity of the system. These findings were in line with the results of Pang et al. (2019).

**TABLE 4:** Effect of enrichment with HA extracted from rooster comb on viscoelastic parameters from the frequency sweep test of the stirred‐type yogurt during storage at 4°C and 1 Hz.

Storage time (day)	Treatments (% (w/v))	*G*′ (Pa)	*G*″ (Pa)	η* (Pa·s)	tan *δ*
1	Control	151.75 ± 12.1^B^	37.86 ± 3.55^B^	24.89 ± 2.73^B^	0.24 ± 0.00^B^
	0.8	496.11 ± 32.26^A^	111.15 ± 10.43^A^	80.91 ± 9.02^A^	0.22 ± 0.04^C^
	0.9	93.56 ± 9.08^C^	24.18 ± 1.01^C^	15.38 ± 1.62^C^	0.25 ± 0.01^A^
21	Control	103.53 ± 8.12^C^	24.98 ± 2.12^C^	16.95 ± 1.51^C^	0.24 ± 0.01^A^
	0.8	584.87 ± 41.22^A^	116.98 ± 10.18^A^	94.93 ± 9.23^A^	0.20 ± 0.00^C^
	0.9	345.84 ± 28.81^B^	81.04 ± 7.03^B^	56.53 ± 4.72^B^	0.23 ± 0.01^B^

*Note:* Values are mean ± standard deviation in triplicate. Means are compared only within a column; mean values with different capital letters are statistically significant differences at the probability level of 95%.

### 3.5 Effect of RCE on Sensory Properties

**FIGURE 4:**
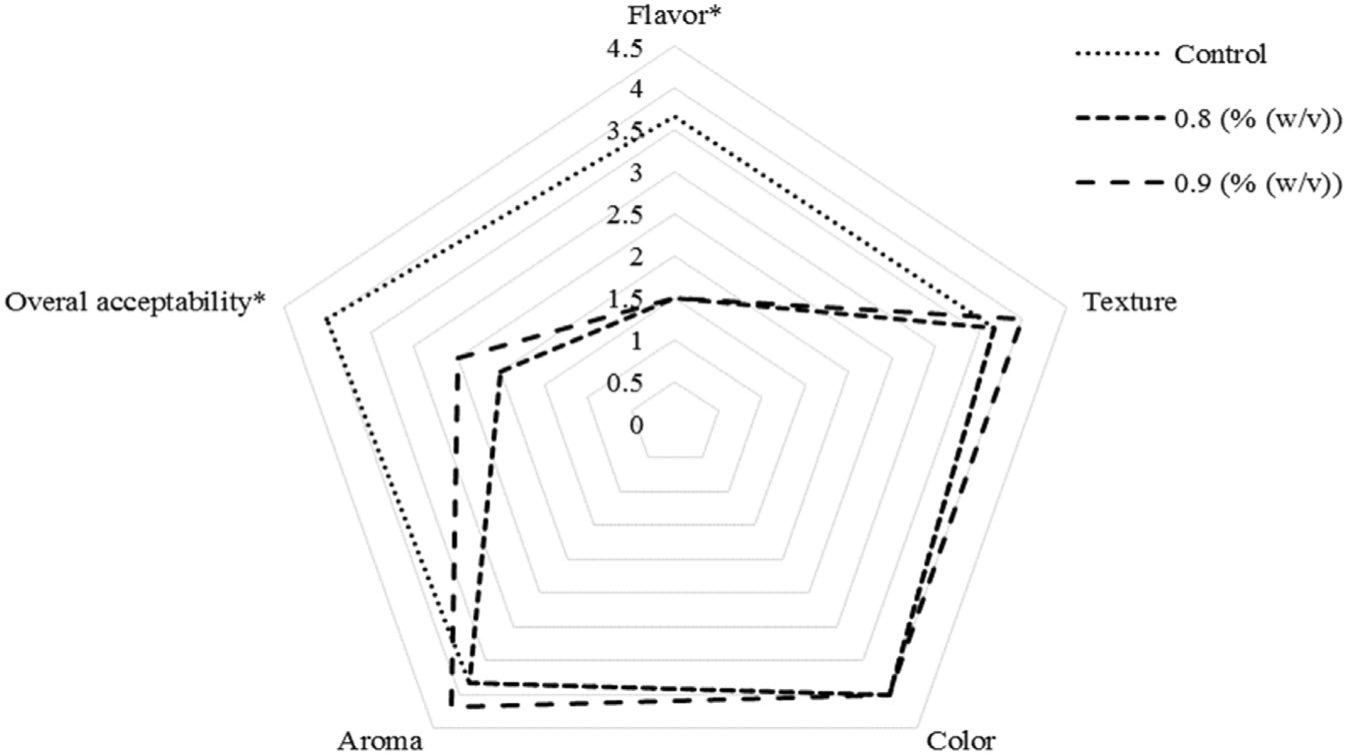
Effect of enrichment with HA extracted from rooster comb on the sensory properties of the stirred yogurt. *Statistically significant differences at the probability level of 95%.

Sensory properties are crucial in evaluating novel food formulations. When enriching food, the type and concentration of nutrients, as well as the choice of carrier, must be carefully considered. Factors like availability, taste, nutrient content, and economic feasibility influence the selection of food‐enriching sources (Zhang et al. 2023). The overall properties of fermented dairy products, including sensory and nutritional aspects, are affected by the milk's chemical composition, processing conditions, added flavorings, and the activity of starter bacteria during fermentation (Sharma et al. 2023). The sensory characteristics of the samples, with or without RCE, are shown in Figure 4. The flavor and overall liking of the stirred yogurt without RCE were significantly higher than those of the RCE‐enriched samples (*p* < 0.05, 21st day). Sensory evaluators gave the control sample higher scores for these properties. However, no significant differences were observed in texture, color, or aroma between the enriched and control samples (*p* > 0.05, Figure 4). These results may be due to the presence of glycosaminoglycans, free amino acids, proteins, and fibers in RCE, which reduced the aroma intensity in the treated samples (Ramos‐Peralonso 2013). It is assumed that the increased viscosity (*η*) of the enriched samples slows the movement of volatile compounds and flavoring ingredients. As a result, fewer of these molecules are released in the mouth, which may slightly affect the flavor (Milani and Koocheki 2011). The decrease in the overall acceptance of samples with varying concentrations of RCE compared to the control may result from the weakening of the casein network under acidic conditions. Additionally, the proteolysis of protein molecules leads to greater whey loss and lower consistency in the final products. Similar results were reported by Sandoval‐Castilla et al. (2004), Bhat, Deva, and Amin (2018), and Mousavi et al. (2019) in gel‐based systems containing modified tapioca starch, fruit fiber, low methoxy pectins, and flaxseed, respectively. In contrast, Hamad et al. (2017) noted that fortification with HA‐producing bacteria positively affected the sensory and functional properties of yogurt as a dietary supplement.

## 4 Conclusions

Yogurt containing RCE demonstrated a notable reduction in pH and microbial load, alongside an increase in acidity during storage. Treatments with 0.8 g of RCE exhibited lower syneresis, brightness, and setting time. Additionally, the enriched yogurts displayed thixotropy and pseudoplasticity, with the Herschel–Bulkley model effectively fitting the experimental data. *G*′ consistently exceeded *G*″ in all samples, confirming the solid structure of the products. However, the enriched samples received lower sensory scores for flavor and overall acceptability, while differences in texture, color, and aroma compared to the control were insignificant. In conclusion, the results indicate that RCE could serve as a natural stabilizer in stirred yogurt, offering a viable alternative to synthetic options. Further research is needed to investigate its potential applications in the commercial formulations of other dairy products.

## Author Contributions

**Nastaran Tabari Shahandasht:** formal analysis (equal), investigation (equal), methodology (equal), software (equal), writing – original draft (equal). **Marzieh Bolandi:** conceptualization (equal), data curation (equal), investigation (equal), supervision (equal), writing – review and editing (equal). **Majid Rahmati:** data curation (equal), investigation (equal), validation (equal). **Moslem Jafarisani:** data curation (equal), investigation (equal), validation (equal).

## Ethics Statement

The study was reviewed and approved by the Islamic Azad University of Damghan branch, and informed consent was obtained from each subject prior to their participation in the study.

## Conflicts of Interest

The authors declare no conflicts of interest.

## Data Availability

Available data will be expressed on request.
